# Serum Inflammatory Biomarkers in the Diagnosis of Periprosthetic Joint Infections

**DOI:** 10.3390/biomedicines9091128

**Published:** 2021-09-01

**Authors:** Irene K. Sigmund, Stephan E. Puchner, Reinhard Windhager

**Affiliations:** Department of Orthopaedics and Trauma Surgery, Medical University of Vienna, Spitalgasse 23, 1090 Vienna, Austria; stephan.puchner@meduniwien.ac.at (S.E.P.); reinhard.windhager@meduniwien.ac.at (R.W.)

**Keywords:** periprosthetic joint infection, diagnosis, serum inflammatory markers, biomarker, CRP, fibrinogen, leukocyte count, differential, platelet count to mean platelet volume ratio, percentage of neutrophils, neutrophils to lymphocytes ratio, D-dimer, interleukin 6, procalcitonin

## Abstract

Accurate preoperative diagnosis of periprosthetic joint infections (PJIs) can be very challenging, especially in patients with chronic PJI caused by low-virulence microorganisms. Serum parameters, such as serum C-reactive protein (CRP) or the erythrocyte sedimentation rate (ESR), are—among other diagnostic test methods—widely used to distinguish septic from aseptic failure after total hip or knee arthroplasty and are recommended by the AAOS in the preoperative setting. However, they are systemic parameters, and therefore, unspecific. Nevertheless, they may be the first and occasionally the only preoperative indication, especially when clinical symptoms are lacking. They are easy to obtain, cheap, and are available worldwide. In the last decade, different novel serum biomarkers (percentage of neutrophils, neutrophils to lymphocytes ratio, platelet count to mean platelet volume ratio, fibrinogen, D-Dimer, Il-6, PCT) were investigated to find a more specific and accurate serum parameter in the diagnosis of PJI. This article reviews the diagnostic value of established (serum CRP, ESR, WBC) and ‘novel’ serum inflammatory biomarkers (fibrinogen, D-dimer, interleukin-6 (IL-6), procalcitonin, percentage of neutrophils (%N), neutrophils to lymphocytes ratio (NLR), platelet count to mean platelet volume ratio (PC/mPV)) for the preoperative diagnosis of periprosthetic joint infections.

## 1. Introduction

To find the optimal surgical therapy (DAIR (debridement, antibiotics, irrigation, and implant retention), one-stage, two-stage revision) for eradicating a periprosthetic joint infection (PJI), an accurate diagnosis is essential. Serum inflammatory parameters were recommended by the American Association of Orthopaedic Surgeons (AAOS) to aid in the preoperative diagnosis of PJI [1]. They may be the first and occasionally the only preoperative indication, especially when clinical symptoms (for example, redness, swelling, joint effusion) are lacking. They are easy to obtain, cheap, and are available all over the world. However, they are systemic parameters, and therefore, unspecific [2]. They need to be complemented by more specific diagnostic test methods, such as synovial fluid analysis, microbiology and histology of deep tissue samples, and sonication fluid analysis. However, while synovial fluid analyses are easily to perform preoperatively, sampling of deep tissue and sonication fluid is invasive, their analysis is time-consuming, and results are only available postoperatively. Hence, these latter two test methods have no preoperative value for diagnosing PJI and for planning the optimal therapy preoperatively. Although unspecific, serum biomarkers can provide preoperative information and represent, therefore, an important screening tool.

At the International Consensus Meeting (ICM) in 2018, elevated serum C-reactive protein (CRP), serum erythrocyte sedimentation rate (ESR), and serum D-dimer were defined as minor criteria in the revised ICM criteria [3]. The European Bone and Joint Infection Society (EBJIS) integrated serum CRP as a single serum parameter in their PJI definition as suggestive criterion in their “infection likely” group [4], while the Infectious Diseases Society of America (IDSA) does not include any serum biomarker in their definition [5].

In the last decade, different novel serum biomarkers were investigated to find a more specific and accurate serum parameter.

In this review article, we discuss the performance of established (serum CRP, ESR, WBC) and ‘novel’ serum inflammatory biomarkers (fibrinogen, D-dimer, interleukin-6 (IL-6), procalcitonin, percentage of neutrophils (%N), neutrophils to lymphocytes ratio (NLR), platelet count to mean platelet volume ratio (PC/mPV)) in the preoperative diagnosis of periprosthetic joint infections. We provide a comprehensive overview of the current literature and arising controversies and try to elucidate the impact of each single serum parameter on the preoperative diagnosis of PJI.

## 2. C-Reactive Protein (CRP)

Serum C-reactive protein (CRP) is an acute-phase reactant produced by the liver in response to infection, inflammation, and neoplasm [6,7]. CRP is a general parameter affected by any systemic inflammatory disease (autoimmune disorders, coronary heart disease, active cancer, acute or chronic renal failure, obesity, infections other than joint). Therefore, it is not purely specific for PJI. However, it is still used as an established inflammatory parameter in the preoperative diagnosis of periprosthetic joint infections [3,8], and the AAOS and the MSIS support its use in the preoperative setting [1,9].

In the literature, a broad range of sensitivities from 62% to 100%, and specificities from 64% to 96% [2,6,10,11,12,13,14,15,16,17,18,19,20,21,22,23,24,25,26,27,28,29] were described. Table 1 shows the results of the recent literature of serum CRP depending on the used infection definition. A proper comparison between studies published before the introduction of infection criteria in 2011 [30] is difficult due to various infection definitions (classification bias). Nilsdotter-Augustinsson et al. only used significantly positive cultures (incubation time: 10 days) together with the clinical evaluation by orthopedic surgeons [10]. The results of more accurate diagnostic methods (for example, histopathology, synovial fluid leukocyte count and differential, the presence of a sinus tract) were not considered in their infection classification. Hence, some infections, especially low-grade infections, may be misdiagnosed as aseptic failure, leading to false accuracies. The same applies for the studies by Bottner et al. [11], Ghanem et al. [6], and Piper et al. [6] in which some diagnostic methods were not taken into account in their applied infection definitions (Table 1). In addition, different thresholds of serum CRP levels with a wide variety (3.0–32.0 mg/L) have been proposed, making a comparison of the performances between the different studies problematic. Nevertheless, a general cut-off of >10 mg/L is recommended by the EBJIS [4], while at the ICM in 2018, a distinction between acute and chronic infections was made, with cut-offs of >100 mg/L and 10 mg/L, respectively. However, the authors stated in their article that these criteria were never validated on acute infections [3]. Possible explanations of the various accuracies and thresholds of CRP are the different infection definitions used, the heterogenous spectrum of microorganisms detected by the different study groups (low- and high-virulence pathogens), the dissimilar incubation period (5 to 14 days), patient factors (autoimmune disorders, cancer, age, sex, underlying diseases, medications, etc.), and the influence of antimicrobial or immunomodulatory therapies (corticosteroids).

Periprosthetic infections can be classified as acute or early postoperative (31%), chronic late (56%), and acute hematogenous (13%) infections [31]. Acute postoperative and acute hematogenous infections, which are commonly caused by high-virulence organisms, such as *Staphylococcus aureus* or streptococci, can usually be easily identified due to their clinical presentation (drainage, fever, redness, massive joint effusion, etc.), joint aspiration (pus, leucocyte count), and extensive immune response [32,33,34]. Serum CRP levels are highly elevated in these acute planktonic infections. However, in the immediate postoperative setting, the serum CRP levels are affected by tissue injuries during the operation. The levels can remain increased for approximately 30 to 60 days after surgery, limiting its diagnostic value in this setting [7,35]. False-positive results can lead to a potential overtreatment, including unrequired aggressive revision surgery and prolonged antimicrobial therapy.

In late-chronic infections, low-virulence microorganisms are capable of forming biofilm. Bacteria can live dormant in this self-made environment without interacting with inflammatory cells of the host immune system, resulting in a limited release of inflammatory serum biomarkers, including serum CRP [36,37]. In our recently published study, 46 (61%) of the 75 PJI patients were culture positive, including 14 infections caused by low-virulence (30%) and 32 by high-virulence microorganisms (70%) [2]. The median serum CRP levels differed significantly between these groups (17.6 mg/L (IQR 9.5–36.9), 49.2 mg/L (IQR 10.9–231.9), respectively; *p* = 0.044), with lower values in PJI cases caused by low-virulence pathogens. In addition, Ettinger et al. showed lower CRP levels in patients with PJI caused by low-virulence organisms (12 mg/L) in comparison with high-virulence bacteria (35 mg/L) [18]. However, patients with any inflammatory comorbidity were excluded in their study. Hence, their results may not be generalizable in clinical practice (selection bias). Perez-Pietro et al. [33] reported that some patients with chronic and low-grade infections would never have been detected when using the AAOS guidelines (in which they concluded that in cases with normal CRP, an infection is unlikely and no further tests are needed) [38]. In their study, 23 of 73 culture-positive chronic infections (32%) showed a normal serum CRP level (cut-off >8 mg/L) preoperatively. In 70% of these 23 patients, a low-virulence microorganism (mainly coagulase-negative staphylococci and *P. acnes*) was identified. However, they included 13 patients with total shoulder and one patient with a total elbow arthroplasty. Additionally, they also excluded patients with rheumatic disease (selection bias). Hence, a proper comparison cannot be done. Whereas Akgün et al. [34] included only hips and knees and did not exclude patients with inflammatory arthropathy in their study evaluating serum CRP levels in 215 culture-positive PJI patients. Nevertheless, they also demonstrated lower median serum CRP levels in those with chronic PJI (10.6 mg/L) than patients with acute postoperative (83.7 mg/L) and acute hematogenous infections (149.4 mg/L) (*p* < 0.001). In 77 patients (35.8%), a normal preoperative serum CRP concentration (<10 mg/L) was observed. In 66 of these patients (85.7%), at least one low-virulence organism was isolated. Lower median levels were described in *Proprionibacterium* spp. (5.4 mg/L), coagulase-negative staphylococci (12.2 mg/L), and *Enterococcus faecalis* infections compared with *Staphylococcus aureus* (194 mg/L) and streptococci (89.3 mg/L) infections (*p* < 0.001).

Hence, low serum CRP levels cannot exclude PJI as stated by the AAOS, especially when caused by low-virulence organisms. These results highlight the false-negative rate of serum CRP in diagnosing PJI. Among immunomodulatory and antimicrobial therapies that can influence the host immune system, the low-virulence and the biofilm-forming properties of organisms may be one of the causes why the sensitivity of serum CRP is low in diagnosing PJI. An infection may be misdiagnosed as aseptic failure due to the lack of a proper immune response.

On the other hand, false-positive cases were also shown, representing the low specificities reported in the literature (Table 1). These can be attributed to the fact that serum CRP is a systemic inflammatory protein, which can be elevated in patients with other systemic inflammatory conditions, such as autoimmune disorders (rheumatoid arthritis, systemic lupus erythematosus, psoriasis, etc.), infections at a site other than joints (pneumonia, bronchitis, infected intravascular devices, urinary tract infections, etc.), and/or active cancer.

In the literature, different thresholds were reported between hips and knees. Alijanipur et al. [14] analyzed the serum CRP concentration in 1095 revision total hip arthroplasties and 594 revision total knee arthroplasties. In the PJI as well as in the aseptic group, a higher median CRP value (PJI: 133 mg/L; aseptic: 7 mg/L) was calculated in knees compared with hips (PJI: 73 mg/L, *p* = 0.02; aseptic: 6 mg/L, *p* < 0.001). The authors explained these findings by the more intense inflammatory reaction to arthroplasty in knees compared with hips. Larsson et al. [39] reported that a total knee arthroplasty procedure is more traumatic to bone and marrow tissue containing more inflammatory cells. Nevertheless, the exact cause is currently unknown.

In patients with a late chronic infection, higher CRP levels were shown in knees (*p* = 0.005), whereas in early postoperative infections, no difference between both joints was reported [14]. In hips, a lower median value was calculated in late chronic infections (56 mg/L) compared with early postoperative infections (143 mg/L, *p* < 0.001). Similar results were described in revision total knee arthroplasties. Therefore, they concluded that different cut-offs in early postoperative and late-chronic infections and between hips and knees might be required. Nevertheless, the study group also excluded patients with comorbidities confounding CRP (selection bias). No distinction between acute hematogenous and chronic infections was done. Hence, a lower median serum CRP level might be possible if only chronic infections were included in this group. Another limitation is the modified infection definition used. They did not perform histological and synovial fluid analysis, which might lead to accuracy changes. Finally, the overall cut-offs were higher compared with the conventional threshold of 10 mg/L. Further studies are needed to elucidate this topic more precisely.

Although serum CRP is an inexpensive and convenient parameter, preoperative serum CRP results should be interpreted with caution. The surgeon needs to be aware of possible influences that can affect its concentration. Hence, it cannot be utilized as confirmatory criterion in the diagnosis of PJI but can be recommended as a suggestive diagnostic method in the preoperative setting of a revision hip or knee arthroplasty. It needs to be complemented by more accurate diagnostic methods (such as synovial fluid analysis, histology, microbiological analysis).

## 3. Erythrocyte Sedimentation Rate (ESR)

If an infection is suspected, the erythrocyte sedimentation rate (ESR) is a commonly used blood parameter. For ESR assessment, the reaction time in which the red blood cells settle near the bottom of the test tube is measured [40]. Following primary arthroplasty, ESR increases until reaching a peak after 5 to 7 days postoperatively and constantly decreases to normal levels in 3 to 12 months [7]. ESR rises and drops more slowly and less prominently than serum CRP [39,41]. However, it is—like serum CRP—a general parameter affected by any systemic infection or inflammation. Therefore, it is an unspecific marker for PJI. Nevertheless, the AAOS and the MSIS again endorse the use of ESR to aid in preoperative diagnosis of PJI [1,9], while the EBJIS does not include it in their infection definition due to their low accuracy. In the literature, sensitivities ranged from 33% to 95%, and specificities from 60% to 100% [6,10,11,12,14,15,19,21,22,23,24,26,27,28,29,42]. Table 2 shows the recent literature of ESR depending on the used infection definition. Here, again, different infection criteria were used among the listed studies. The authors of the itemized studies also calculated various optimal thresholds between 13 mm/h and 46 mm/h (Table 2) for diagnosing PJI while the ICM criteria (2018) suggest the conventional cut-off of 30 mm/h in chronic periprosthetic joint infections. The diversity of infection definitions, microorganisms, incubation periods, patient factors, and patient medication may have led to these differences in accuracies and thresholds of ESR. In addition, the technical details (measuring methods, type of collection tube, mixture technique) of ESR were not consistently provided in these studies. This lack of information complicates a proper comparison.

Due to a proposed increase of accuracies, a combination of ESR and serum CRP was recommended [6,43]. Spangehl et al. [43] concluded that the combination of ESR and CRP is reliable for predicting the absence of infection after analyzing a cohort of 178 patients with 202 revision total hip arthroplasties. Ghanem et al. [6] reported an increase of sensitivity (98%) when ESR (>30 mm/h) or CRP (>10 mg/L) was elevated compared with one parameter alone (ESR: 94%; CRP: 91%); however, specificity decreased to 59% (ESR: 70%; CRP: 77%). If CRP as well as ESR needed to be elevated, sensitivity decreased to 88%, but specificity increased to 88%. Therefore, it seems that the overall accuracy does not change, leading to the assumption that the combined assessment has no benefit in the diagnosis of PJI.

Alijanipour et al. [14] showed in revision total hip arthroplasties a lower sensitivity and specificity when CRP and ESR was combined (86% and 61%, respectively) compared with ESR alone (95% and 71%, respectively). In revision total knee arthroplasty, sensitivity was nearly similar (ESR + CRP: 96%; ESR: 94%), and specificity decreased (ESR + CRP: 54%; ESR 68%). These findings highlight our above-mentioned assumption that a combination does not add value to the preoperative diagnosis of PJI.

Besides this, Alijanipour et al. [14] also showed higher ESR values in late-chronic knees compared with late-chronic hips (*p* = 0.005), but no difference between both joints was reported in early postoperative PJIs. Wu et al. [27] also compared hips and knees, showing lower sensitivities of ESR (cut-off 29 mm/h) in hips (58%) than in knees (76%). However, no proper comparison between sensitivities or AUCs was done. Specificities were nearly similar between both joints (hips: 91%; knees: 94%). However, less data exists regarding different thresholds between revision total hip and knee arthroplasties. More studies are needed to elucidate this topic.

ESR levels might be normal in patients with PJI caused by low-virulence organisms [19]. In a study by Perez-Pietro et al. [33], 17 of 23 culture-positive PJIs (23%) with a normal serum CRP showed normal ESR levels (cut-off >34 mm/h) preoperatively. Hence, they concluded that ESR might be of little value as a diagnostic test in PJI and stated that ‘diagnostic criteria which use ESR and CRP might misdiagnose up to one fourth of PJI’.

Although ESR shows low accuracies, it is generally used as a screening tool due to its simplicity and cost-effectiveness [44,45]. Nevertheless, it may be used as a suggestive test, but it is not recommended as a confirmatory criterion in diagnosing PJI.

## 4. White Blood Cell Count (WBC)

The serum white blood cell count (WBC) is used in diagnosing many different infections [46]. Therefore, it is also commonly ordered preoperatively in suspected PJI cases. However, in diagnosing PJI, it shows poor sensitivities (21–42%) but good specificities (89–94%) when cut-off levels between 8 and 10 G/L were used [2,16,23,25]. Lower cut-off values were described by Glehr et al. [13] (cut-off: 5.48 G/L) and Klim et al. [20] (cut-off 5.68), leading to a higher sensitivity (91% and 90%, respectively) but at the expense of specificity (34% and 39%, respectively). Table 3 shows the recent literature of WBC depending on the used infection definition [2,10,11,13,15,16,17,20,23,25,29,35].

There is inconsistent data about the value of preoperative WBC for distinguishing septic from aseptic revision arthroplasties. Some studies showed that the serum WBC levels were not higher in infected patients compared with noninfected patients [10,11,47,48,49]. Nilsdotter et al. [10] reported that the median WBC before surgery was in the normal range. In the study by Bottner et al., 14 of 21 PJI cases (67%) had normal WBC levels (cut off: 6300 G/L). No difference was calculated between septic and aseptic groups (*p* = 0.086). Randau et al. [16] also did not find a difference between PJI, aseptic, and control cases when using ANANOVA analysis. In addition, only 20% of PJI patients presented with an elevated WBC.

On the other hand, some studies found that the WBC values are significantly higher in patients with PJI compared with aseptic failure cases [13,15,17,20,23,29,35,50]. Toossi et al. [35] observed a mean WBC of 9236 cells/µL (95% CI 8896–9575 cells/µL) in infected cases and 7331 cells/µL (7204–7458 cells/µL) (*p* < 0.001) in noninfected cases. In the study by Elgeidi et al. [15], the mean WBC in PJI patients was 12.3 G/L and in aseptic failure patients, it was 7.7 G/L (*p* < 0.0001). In knees (septic: 13.5 G/L, aseptic 7.9 G/L) and in hips (septic: 11.6 G/L, aseptic: 7.6 G/L), a higher mean was shown in septic compared with aseptic cases (*p* = 0.001 and *p* = 0.0003, respectively). Yang et al. [29] also reported in a cohort of 156 patients, including 57 PJIs and 99 non-PJIs, a difference between both groups regarding serum WBC (PJI: 7.8 G/L; non-PJI: 6.4 G/L; *p* < 0.001). In our study [2], the mean WBC in septic cases (*n* = 75) was 8.8 G/L and in aseptic patients (*n* = 102), it was 7.0 G/L (*p* < 0.0001). In early PJI cases (<90 days), Yu et al. [23] also demonstrated a higher median value of WBC in infected (8.2 G/L) compared with uninfected (6.1 G/L) cases (*p* = 0.0024). However, most of the reported WBC values (in the latter four studies) were less than the conventionally used cut-off of 10 G/L in infected cases, leading to the assumption that a lower cut-off may be more accurate in diagnosing PJI. Nevertheless, lower cut-offs (between 5–7 G/L) also showed poor accuracies in the literature (sensitivities and specificities: 5.48 G/L: 91% and 34%; 5.68 G/L: 90% and 39%; 6.27 G/L 80% and 48%; 6.58 G/L: 81% and 59%) [13].

Additionally, other serum parameters showed better performances in comparison to serum WBC in the diagnosis of PJI [7,15,17]. Berbari et al. [7] reported in their metanalysis that interleukin-6 (IL-6) and CRP had a higher odds ratio than serum WBC for discriminating septic from aseptic cases. Elgeidi et al. [15] could confirm the better performance of IL-6 in comparison to WBC (*p* = 0.0001), and Randau et al. [16] demonstrated higher accuracies of serum CRP (AUC: 0.83) and procalcitonin (PCT; AUC: 0.85) than WBC (AUC: 0.63: *p* = 0.002; *p* = 0.006).

Nevertheless, due to the low accuracies reported in the literature, serum WBC may not be helpful to aid in the preoperative diagnosis of PJI. Hence, it should only have a limited role in routine clinical workup of patients with suspected PJI.

## 5. Percentage of Neutrophils (%N)

The percentage of neutrophils (%N) has been shown to be a laboratory predictor for periprosthetic joint infections [2,35]. Tossi et al. [35] analyzed 1856 revision surgeries (1543 patients) compromised of 751 PJI cases and 1105 aseptic failures. They reported a mean %N of 63% (95% CI: 62–64%) in the aseptic and 69% (68–70%) in the septic group (*p*< 0.001). The best threshold was found to be 69% for neutrophil percentage with an only moderate sensitivity of 53% (48–58%), specificity of 75% (72–78%), and AUC of 0.652 (0.623–0.679). In our recently published study [2], we found nearly similar results. The mean %N in septic cases was 72% (range: 54–94%) and in aseptic cases, it was 65% (41–90%) (*p* < 0.0001). The optimal cut-off in our cohort was also 69% with again an only moderate sensitivity of 66% and specificity of 67%. The %N showed a worse performance in comparison with serum CRP (*p* < 0.0001) and fibrinogen (*p* < 0.0001).

Nevertheless, only few studies are available to date that have investigated the performance of %N. More studies are needed to evaluate the accuracy of this parameter. However, due to the listed studies (Table 3), this test shows an inferior value in the diagnosis of PJI and cannot be recommended in daily clinical routine use at this stage.

## 6. Neutrophils to Lymphocytes Ratio (NLR)

The neutrophils to lymphocytes ratio (NLR) is commonly used to predict outcomes in oncology and cardiovascular diseases [51,52]. It also showed promising results in the diagnosis of infection. During states of bacterial infection, the neutrophil count increases and lymphocytes decrease [53,54]. Josse et al. [55] reported that NLR (>2.3) may be an independent predictor for major complications (for example, wound infection) preoperatively in patients receiving colorectal surgery. In a study by Bolat et al. [56], NLR was a valid predictor for early postoperative infections in patients undergoing penile prosthesis implantation. DeJager et al. [57] showed that NLR (0.73) had a better AUC than serum CRP (0.62) in predicting bacteremia. Additionally, Qu et al. [58] demonstrated good results in predicting blood stream infections. Following posterior lumbar spinal surgery, Shen et al. [59] showed a good predictive value of NLR for surgical site infections.

Yombi et al. [60] reported that NLR may be a potentially better serum parameter for diagnosing early PJIs because it showed a faster normalization in comparison with CRP after total knee arthroplasties. Yu et al. [23] analyzed NLR in a cohort of 121 cases, including 20 early PJIs and 101 aseptic cases. The median NLR was significantly higher in PJIs (5.2) than in uninfected cases (2.1; *p* < 0.001). NLR (cut-off: 2.13) showed a higher AUC (0.802) with a sensitivity of 85% and specificity of 68% than CRP (0.793). However, IL-6 had the highest AUC in their cohort followed by NLR, CRP, ESR, and WBC. Nevertheless, no statistical comparison was done between AUCs. While they concluded that NLR may be considered as a useful parameter, we cannot recommend it in clinical routine use due to its low accuracy. In our study evaluating 177 patients (75 PJIs) [2], the optimal threshold was 3.82 with only moderate sensitivity (63%) and specificity (73%). Although NLR was higher in septic patients (mean: 4.0, range: 1.0–44.7; aseptic: 3.0, range: 0.9–18.1; *p* = 0.001), serum CRP and fibrinogen showed better performances than NLR (*p* < 0.0001). However, Yu et al. only investigated early postoperative PJIs while we also included late chronic infections. This might possibly explain the different results.

Although NLR is a cheap and convenient serum parameter, the overall accuracies were low and thus it cannot be considered as a sufficient marker in the diagnosis of PJI.

## 7. Platelet Count to Mean Platelet Volume Ratio (PC/mPV)

The platelet count (PC) and mean platelet volume (mPV) are, due to their easily accessible nature, two frequently ordered coagulation parameters prior to revision surgery. In the presence of inflammation and infection, the platelet production is intensified, and the mean platelet volume drops as megakaryocytes are subject to higher concentrations of circulating thrombopoietin [42,61]. These contrary patterns of PC and mPV leads to an elevated ratio between these two variables. In bacterial infections, platelets are mechano-scavengers that can collect and bundle microorganisms in a way that supports the leukocyte function, and hence, directly facilitate the host’s response to infection [62].

Paziuk et al. [42] were the first authors to assess the performance of preoperative PC/mPV ratio for distinguishing septic from aseptic failure in patients with revision total hip and knee arthroplasty. In 4939 cases, 949 patients were diagnosed with chronic PJI based on the MSIS criteria. However, a serious limitation is the lack of histopathological analysis in this study (some infections could be missed). Nevertheless, PC/mPV was 33.45 in PJI cases compared with 25.68 in aseptic cases. A statistically significant difference was shown between the latter two groups (*p* < 0.001). The optimal threshold for PC/mPV to differentiate between PJI and non PJI patients was 31.7 with a sensitivity of 48% (95% CI: 45–51%) and specificity of 81% (80–82%) (Table 3). The specificity was higher compared with those of CRP (74%) and ESR (78%), while sensitivity was substantially lower (CRP: 79%; ESR: 79%), leading to better AUCs of CRP (0.872) and ESR (0.851) than PC/mPV (0.69). The combination of ESR and CRP (0.8749) and the combination of ESR, CRP, and PC/mPV (0.8768) showed minimal higher AUCs in comparison to ESR or CRP alone but no statistical analysis comparing the AUCs was done.

In our study, the mean PC/mPV values were again different between septic (25.1 (range: 10.7–97.5)) and aseptic (23.1 (9.5–54.9); *p* = 0.001) cases. The optimal threshold of this parameter was calculated, with 29.4 resulting in a low sensitivity of 43% (95%CI 32–54%) and moderate specificity of 81% (72–88%). The combination of CRP and fibrinogen (*p* < 0.0001) and the combination of CRP, fibrinogen, and PC/mPV (*p* = 0.016) were superior to PC/mPV alone. However, the combination of CRP and fibrinogen was not superior to one method alone (CRP: *p* = 0.200, fibrinogen: 0.437).

Huang et al. [28] compared the PC/mPV values in patients with PJI (*n* = 47), patients with primary osteoarthritis (OA; *n* = 64), and patients with aseptic failure (*n* = 38). The median PC/mPV was 35.6 for PJI cases, 25.0 in the OA group, and 25.2 in the aseptic group. There was a statistically significant difference between the PJI group and the OA group (*p* < 0.001) and between the PJI group and the aseptic group (*p* < 0.001). No difference was shown between the OA and the aseptic group (*p* = 0.933). A cut-off of 31.7 showed again a low sensitivity of 55% and moderate specificity of 81%. PC/mPV (0.686) had the lowest AUC compared with CRP (>10 mg/L; AUC: 0.892), ESR (>30 mm/h; AUC: 0.888), plasma fibrinogen (>4.01 µg/mL; AUC 0.873), and plasma D-dimer (>1.17 mg/L; AUC 0.83).

Although PC/mPV showed a moderate to good specificity and is associated with pathophysiological states of infection, it is not a perfect preoperative test for PJI due to a significantly lower sensitivity and accuracy compared with established serum parameters. Hence, PC/mPV cannot be recommended as a serum parameter in diagnosing PJI.

## 8. Fibrinogen

Fibrinogen is synthesized in liver cells and routinely ordered preoperatively for coagulation analysis. A close correlation between the coagulation cascade and the inflammatory mechanism was shown in the literature [63,64]. For example, fibrinogen was correlated with infections, such as sepsis [65], periodontitis [66], and appendicitis [67]. It influences the inflammation process by activating different immune cells [68] and by inducing the synthesis of proinflammatory cytokines, such as interleukin 6 and tumor necrosis factor α, in mononuclear cells [69].

Klim et al. [20] analyzed preoperative fibrinogen levels in a cohort of 84 patients with revision total joint arthroplasties. In total, 55 patients were diagnosed with PJI and 29 as aseptic failures based on modified MSIS criteria (2011). Contrary to the original MSIS criteria, they did not include the synovial fluid white blood cell count and percentage of polymorphonuclear neutrophils; hence, some infections may have been missed. In addition, they also included patients in between stages with spacer exchange (*n* = 5) or reimplantation (*n* = 3), which are known to affect the levels of serum parameters [50]. Nevertheless, when a cut-off level of 519 mg/dL was used, a nearly similar sensitivity (90%) and specificity (66%) for fibrinogen was shown in comparison with CRP (cut-off 11 mg/L; 90% and 74%, respectively). In another study by Klim et al. [25], a cut-off of 515 mg/dL revealed a sensitivity of 94% and specificity of 73%. Increased fibrinogen levels correlated with PJI (*p* < 0.05). In their biomarker model of CRP, fibrinogen, the ratio of fibrinogen to CRP, and the ratio of thrombocytes to CRP, a lower sensitivity (72%) but higher specificity (91%) was shown in comparison with fibrinogen alone. Hence, the diagnostic accuracy did not improve by combining multiple markers.

Bin et al. [26] also showed a good performance of fibrinogen in the diagnosis of chronic PJI in a cohort of 94 revision hip and knee arthroplasties (53 PJI, 37 non-PJI cases). When using a threshold of 360 mg/dL, fibrinogen had the highest AUC (0.928) compared with CRP (cut-off: 4.9 mg/L; 0.893) and ESR (cut-off: 31 mm/h, 0.925), but a statistical comparison between AUCs was not done. Overall, CRP (94%) showed a higher sensitivity compared with fibrinogen (79%), but fibrinogen (95%) showed a higher specificity compared with CRP (73%) (*p* < 0.05). In PJI cases, the median fibrinogen concentration was statistically significant higher (437 mg/dL (IQR 382–495)) in comparison with non-PJI cases (286 mg/dL (IQR 246–325), *p* < 0.001), which has been confirmed in other studies [2,27,28,29].

Wu et al. [27] reported that fibrinogen (cut-off: 361 mg/dL; AUC 0.868) performed better than D-dimer (0.728) and equally to CRP (0.914) and ESR (0.848). In the hip subgroup, fibrinogen (0.879) and CRP (0.844) had the highest AUCs in comparison with ESR (0.761) and D-dimer (0.543). In the knee subgroup, all evaluated parameters showed good performances according to AUCs. Among those, CRP (0.951) showed the highest area under the curve (ESR: 0.891; D-dimer: 0.801; fibrinogen: 0.852). However, no statistical analysis was done to compare the different AUCs. Huang et al. [28] demonstrated similar results. The AUCs of fibrinogen, CRP, ESR, PC/mPV, and D-dimer were 0.873, 0.892, 0.888, 0.686, and 0.835, respectively. In the study by Yang et al., the AUCs of fibrinogen, CRP, ESR, and WBC were 0.916, 0.901, 0.822, and 0.647, respectively. In the two latter articles, no statistical comparison was done between the AUCs of the investigated markers, and patients with an underlying infection or inflammation were excluded.

In our study, fibrinogen (0.785) and serum CRP (0.785) showed better AUCs than WBC (0.626), %N (0.665), NLR (0.677), and PC/mPV (0.619) when comparison analyses between AUCs were done (*p* < 0.05). The performance of serum CRP was not better than the performance of fibrinogen (*p* = 0.620). We also analyzed the fibrinogen levels in patients with PJI caused by low- and high-virulence organisms [2]. In the low-virulence group, the median fibrinogen level was 499 mg/dL (IQR 409–609) and in the high-virulence group, it was 567 mg/dL (496–758). There was no difference between these groups (*p* = 0.111), leading to the assumption that fibrinogen cannot distinguish between infections caused by low- and high-virulence organisms.

Due to the results described above and in Table 4, fibrinogen is comparable to serum CRP and may aid in diagnosing PJI. However, the reported accuracies are not satisfactory to confirm or exclude PJI. Hence, fibrinogen can only be recommended as suggestive criterion in the diagnosis of PJI.

## 9. D-Dimer

Although D-dimer (a coagulation-related biomarker) showed poor accuracies, it is still commonly used to detect venous thromboembolism in orthopedic patients [70]. It is a fibrin degradation product formed during fibrin clot dissolution by plasmin. This serum parameter is not only increased during disseminated intravascular coagulation, but it may also be elevated in the presence of inflammation and infections [71,72].

Schwameis et al. [72] reported that rapidly increased levels of D-dimer may indicate poor outcome in early (within 3.5 h) bacteremia. It is also recommended as a diagnostic and prognostic parameter for infective endocarditis and mycoplasma pneumonia [73,74]. In patients with rheumatoid arthritis, D-dimer levels are elevated due to the inflamed synovium, which secrets fibrin, resulting in elevated values of serum and synovial fluid D-dimer after degradation of fibrin [75]. Following total joint arthroplasty, D-dimer has its peak on the first operative day (4.5 µg/dL) and returns to baseline levels on the second postoperative day [76]. Interestingly, it slowly increases again and shows a second peak at postoperative week 2, returning to baseline levels by postoperative week 6.

In the diagnosis of PJI, Shahi et al. [19] reported promising results for D-dimer to distinguish septic from aseptic revision arthroplasties. D-dimer was more accurate than the established serum parameters ESR and CRP. A threshold of 850 ng/mL showed a sensitivity of 89% and specificity of 93%. In comparison, the sensitivities of CRP and ESR were 79% and 73% and the specificities were 80% and 78%, respectively. The authors concluded that D-dimer seems to be a better test than CRP and ESR. However, they did not describe AUCs of the investigated parameters in their study and a statistical comparison between these parameters was not done. Additionally, they did not perform histopathological analysis; hence, some infections may be misdiagnosed as aseptic failures. Nevertheless, the median values of D-dimer were significantly higher in the PJI group (*n* = 57; 1100 ng/mL) compared with the aseptic failure group (*n* = 86; 299 ng/mL; *p* < 0.0001). Due to this results, D-dimer was included as minor criterion in the ICM criteria 2018 [3].

In contrast, Fu et al. [21] reported in a small cohort of 15 PJI cases and 15 aseptic failure cases an inferior sensitivity (67%) and specificity (60%) of D-dimer when a cutoff level of >850 ng/mL was used. Xiong et al. [22] concluded that D-dimer did not outperform CRP and ESR in the diagnosis of PJI, but it may be comparable with the latter two serum parameters. The AUCs of D-dimer, CRP, and ESR were nearly similar, calculated as 0.890 (0.814–0.966), 0.831 (0.737–0.926), and 0.838 (0.732–0.944), respectively. A statistical comparison was not done. Nevertheless, the mean D-dimer concentration in the PJI group (*n* = 26; 1953.35 ng/mL) was significantly higher than the mean value in the aseptic failure group (*n* = 54; 336.50 ng/mL; *p* < 0.001). Pannu et al. [77] showed in a cohort of 111 patients with revision total hip or knee arthroplasties a poor specificity of D-dimer (32%) when a cutoff level of >850 ng/mL was used, while sensitivity was good (96%). They concluded that this parameter has a poor accuracy for distinguishing septic from aseptic cases. Qin et al. [24] also demonstrated a good sensitivity (93%) of D-dimer but better specificity (75%) when using a higher threshold of >1170 ng/mL. However, they only investigated chronic periprosthetic joint infections in their study but missed differentiating between chronic-late and acute-hematogenous infections. Hence, the results may be misleading. They also stated that the combination of D-dimer and CRP is highly accurate for diagnosing PJI. While sensitivity was improved (98%), specificity was lower than the specificities of one method alone (42%). Therefore, this combination cannot be seen as accurate but rather highly sensitive.

In a cohort of 165 patients with revision hip and knee arthroplasties, Wu et al. [27] reported that fibrinogen (AUC: 0.868) performed better than D-dimer (0.728). They concluded that D-dimer showed limited value in diagnosing PJI. In their subgroup analyses, D-dimer performed only poor to moderate in revision total knee arthroplasties (sensitivity: 91%, specificity: 58%) as well as in revision total hip arthroplasties (sensitivity: 50%, specificity: 71%).

In a study by Huang et al. [28], only D-dimer (PJI: mean 1.6 mg/L, aseptic: mean; 1.21 mg/L, *p* = 0.086) was not able to distinguish PJI from aseptic failure, while CRP, ESR, PC/mPV, and fibrinogen were (*p* < 0.05).

Due to the above-mentioned results, D-dimer should not be used as a first-line serum parameter in the diagnosis of PJI. While Shahi et al. [19] showed a good performance of D-dimer, most of the other studies showed limited diagnostic value of this biomarker. At present, it cannot be recommended as a screening tool, and it should not replace established serum parameters, such as CRP.

Table 5 shows the recent literature of D-dimer depending on the used infection definition [19,21,22,24,27,28,77].

## 10. Interleukin-6 (IL-6)

Interleukin-6 (IL-6) is a serum biomarker that is released by fibroblasts, endothelial cells, macrophages, monocytes, and T2-lymphocytes in the presence of bacterial infections and associated tissue damage [15,78,79]. It triggers the release of CRP from liver cells, B-cell antibody production, and T-cell differentiation [11]. After primary arthroplasty, its concentration increases quickly, peaking after 3 to 6 h. Due to its short half-life of 15 h, IL-6 rapidly returns to normal concentrations (much faster than CRP) [79].

IL-6 has been reported to have good accuracies in diagnosing PJI (Table 6). In a cohort of 78 patients with revision total knee or hip arthroplasty (21 PJIs), Bottner et al. [11] showed that IL-6 had a very good sensitivity and specificity of 95% and 87%, respectively. CRP had a similar sensitivity (95%) but higher specificity (96%) in this cohort. The combination led to a higher sensitivity (100%) but at the expense of specificity (86%). The authors concluded that CRP and IL-6 are excellent screening tests to detect all infected patients. However, their infection definition was only based on positive intraoperative culture and histopathology, leading to a possible underdiagnosis of PJI cases. Area under the curves were not calculated and a higher threshold for CRP (>s32 mg/L) was used. Hence, a clear interpretation is difficult.

Nevertheless, Elgeidi et al. [15] reported that IL-6 (sensitivity: 100%, specificity: 91%, accuracy: 93%) was the most accurate serum marker among ESR (82%, 83%, 83%), CRP (100%, 86%, 88%), and WBC (91%, 76%, 80%) in a small cohort (*n* = 40; PJI = 11) of patients with revision total joint (knee and hip) arthroplasty. The combination of CRP and IL-6 showed an even higher sensitivity (100%), specificity (99%), and accuracy (98%). Therefore, they also recommended the use of IL-6 and the latter combination in the diagnosis of PJI. However, the number of included patients was small, histopathology was not part of their infection definition, and cultures were only incubated for 7 days. In a study by Yu et al. [23] investigating only early postoperative PJIs (<90 days; *n* = 20) and aseptic failures after total joint arthroplasty (*n* = 101), they concluded that IL-6 (AUC 0.814) was the best serum parameter among NLR (0.802), CRP (0.793), ESR (0.744), and WBC (0.632). However, no statistical comparison between AUCs was done.

In a study by Glehr et al. [13], CRP (AUC 0.90) was generally superior to IL-6 (AUC 0.80). When a cut-off level of 2.55 pg/mL was applied, sensitivity was 94%, but specificity was low (53%). If a threshold of 4.7 pg/mL was used, sensitivity decreased to 86% and specificity increased to 67%. The combination of CRP and IL-6 revealed a sensitivity of 84% and specificity of 68%. These values were lower in comparison with the results of CRP alone (sensitivity: 91%, specificity: 72%). Hence, the combination cannot be recommended based on this data. However, the authors did include patients with spacer exchanges (blood samples were taken during prosthesis-free interval) and, in addition, they did not perform synovial fluid WBC and percentage of polymorphonuclear neutrophils, which may led to a potential underdiagnosis of PJI.

Randau et al. [16] also demonstrated insufficient sensitivities (2.6 pg/mL: 79%, 6.6 pg/mL: 49%) and specificities (2.6 pg/mL: 58%, 6.6 pg/mL: 88%) when cut-offs of 2.6 pg/mL and 6.6 pg/mL were applied.

However, the available data reveals controversial results regarding the performance of IL-6. A proper comparison cannot be made due to the different infection definitions and cut-offs (Table 6) [11,13,15,16,18,23,25,80]; hence, no recommendation can be given. Further studies are needed to elucidate the diagnostic value of IL-6 in diagnosing PJI.

## 11. Procalcitonin (PCT)

Procalcitonin (PCT) showed a good performance in diagnosing sepsis and bacterial infections [81,82,83]. It is the prohormone of calcitonin and is released by C-cells of the thyroid and neuroendocrine cells of the intestines and lungs in the setting of bacteremia [81]. The half-life of PCT is 25 to 30 h and it reaches a peak 6 h after the onset of sepsis [84,85]. The success of antimicrobial therapy is better reflected by the course of PCT concentrations compared with CRP levels [84,86].

In the diagnosis of periprosthetic joint infection, PCT showed limited diagnostic value (Table 7) [11,13,16,17,18,25]. Although Bottner et al. [11] could show a very good specificity (98%) and a statistically significant difference between the mean PCT level in septic (1.5 ng/mL) and aseptic (0.1 ng/mL) cases (*p* = 0.0033), the sensitivity of PCT was poor (33%). Hence, PCT is a specific parameter—an infection is likely in patients with elevated PCT concentrations. These results were confirmed by other authors analyzing the performance of PCT [13,16,25].

Glehr et al. [13] showed that the combination of CRP and PCT had a higher specificity (83%) compared with CRP alone (72%) but at the expense of sensitivity (CRP + PCT: 83%; CRP alone 91%); and the combination of IL-6 and PCT also showed a higher specificity (68%) compared with IL-6 alone (53%) but sensitivity decreased (IL-6 + PCT: 83%; IL-6 alone: 94%). They concluded that CRP had the best performance among the investigated parameters in diagnosing PJI.

In the study by Randau et al. [16], the sensitivity of PCT (13%) was even lower than that of WBC (21%). They also demonstrated that PCT concentrations were only elevated in patients with obvious signs of SIRS, sepsis, and fulminant infection. Ettinger et al. [18] concluded that PCT is not a suitable parameter to differentiate between aseptic failure and low-grade infections.

Although PCT seems to be a good marker to detect systemic bacterial infections, it cannot be used as a preoperative parameter in the diagnosis of periprosthetic joint infections due to its poor accuracy.

## 12. Conclusions

Accurate preoperative diagnosis of periprosthetic joint infections can be very challenging, especially in infections caused by low-virulence organisms.

In this review, we showed that serum parameters in general have insufficient accuracy for diagnosing PJI (Table 8). Serum parameters are systemic parameters and, therefore, unspecific and can be false-positive in patients with other systemic inflammatory conditions, such as autoimmune disorders (rheumatoid arthritis, systemic lupus erythematosus, psoriasis, etc.), infections at a site other than joints (pneumonia, bronchitis, infected intravascular devices, urinary tract infections, etc.), and/or active cancer.

The low sensitivities may be explained by the lack of a proper immune response in patients with chronic encapsulated joint infections caused by low-virulence organisms capable of forming biofilm, patients with a sinus tract, patients with a reduced immune system, and/or patients under the influence of immunomodulatory and antimicrobial therapies.

In addition, the literature shows tremendous variability between studies regarding the chosen infection classification, study population, confounding factors (autoimmune disorders, cancer, age, sex, underlying diseases, medications, etc.), antimicrobial or immunomodulatory therapies, the spectrum of causing microorganisms, dissimilar incubation periods, and the provided information on test reproducibility making a clear comparison and interpretation difficult.

At present, serum CRP and fibrinogen seem to be the serum parameters with the best performances among the presented biomarkers (Table 1, Table 2, Table 3, Table 4, Table 5, Table 6 and Table 7). Although accuracies are limited, we recommend these two parameters in the preoperative diagnosis of PJI as suggestive criteria. However, results should be interpreted with caution in clinical routine use. Elevated serum parameters should raise awareness among clinicians and more specific diagnostic analyses (for example, synovial fluid analysis, etc.) should be initiated.

## Figures and Tables

**Table 1:** Comparison of the literature regarding serum-C-reactive protein (CRP) in the diagnosis of periprosthetic joint infection.

Serum-CRP	Infection Definition	Cut Off (mg/L)	Sensitivity	Specificity
Nilsdotter-Augustinsson et al., ActaOrtho, 2007 [10]	Positive cultures together with the clinical evaluations by orthopedic surgeons	10	82%	71%
Bottner et al., JBJS Br, 2007 [11]	Based on findings of intraoperative culture and histology	32	95%	96%
Ghanem et al., IntJInfDiseases, 2009 [6]	≥1 of the following criteria: (1) an abscess or sinus tract communicating with the joint space, (2) positive preoperative aspiration culture on solid media, (3) ≥2 positive intraoperative cultures or 1 positive culture on solid media in conjunction with the presence of other indicators of infection (gross intracapsular purulence, elevated cell count and differential of the aspirate fluid)	10	91%	77%
Piper et al., PlosOne, 2010 [12]	≥1 of the following criteria: (1) visible pus surrounding the implant, (2) a positive histopathological examination, (3) a sinus tract communicating with the implant, or (4) positive periprosthetic tissue culture and positive sonicate fluid culture for the same microorganism	Knee 14.5 Hip 10.3	79% 74%	88% 79%
Glehr et al., CORR, 2013 [13]	MSIS 2011	10.25	91%	72%
Alijanipour et al., CORR, 2013 [14]	MSIS 2011	Knee 10	97%	70%
		Hip 10	88%	77%
Elgeidi et al., Int Orthop, 2014 [15]	≥1 of the following criteria: visible pus surrounding prosthesis, (2) sinus tract communicating to implant, (3) growth of bacteria on culture (≥2 intraoperative cultures or growth of a virulent organism in a single specimen)	18.0	100%	86%
Randau et al., Plos One, 2014. [16]	≥1 of the following criteria: (1) purulent synovial fluid or ≥1700 leukocytes/µL or ≥ 65% neutrophils in the joint aspirate (TKA) (≥3600 leukocytes/µL or ≥80% neutrophils (THA)), (2) histological confirmation of PJI, (3) pathogen detection in sterile joint aspiration or in at least two intraoperative tissue specimen, or (4) definitive signs of PJI clinically or intraoperatively (e.g., sinus tract)	9.1	62%	83%
Yuan et al., Surgical Infection, 2015 [17]	≥1 of the following criteria: (1) ≥1 positive tissue culture, (2) pus surrounding the prosthesis, or (3) positive histopathology	15	76%	72%
Ettinger et al., CID, 2015 [18]	MSIS 2011	3	80%	64%
Shahi et al., JBJS Am, 2017 [19]	MSIS 2014	10	79%	80%
Klim et al., Sci Rep, 2018 [20]	MSIS 2011	11	90%	74%
Fu et al., JoA, 2019 [21]	MSIS 2011	10	80%	80%
Xiong et al., JOSR, 2019 [22]	MSIS 2011	10	85%	65%
Yu et al., BMC Musculoskeletal Disorders, 2020 [23]	MSIS 2011	9.27	70%	79%
Qin et al., JoA, 2020 [24]	MSIS 2014	7.5	81%	66%
Klim et al., Int Orthop, 2020 [25]	MSIS 2011	10.3	90%	67%
Bin et al., JoA, 2020 [26]	MSIS 2011	4.93	94%	73%
Wu et al., JoA, 2020 [27]	MSIS 2014	10.8	73%	95%
Huang et al., Orthopaedic Surgery, 2021 [28]	MSIS 2014	10	74%	91%
Yang et al., Sci Rep, 2021 [29]	ICM 2018	12.51	91%	83%
Sigmund et al., Int Orthop, 2021 [2]	EBJIS	10	68%	87%

**Table 2:** Comparison of the literature regarding the erythrocyte sedimentation rate (ESR) in the diagnosis of periprosthetic joint infection.

ESR	Infection Definition	Cut-Off (mm/h)	Sensitivity	Specificity
Nilsdotter-Augustinsson et al., ActaOrtho 2007 [10]	Positive cultures together with the clinical evaluations by orthopedic surgeons	30	64%	87%
Bottner et al., JBJS Br, 2007 [11]	Based on findings of intraoperative culture and histology	32	81%	89%
Ghanem et al., IntJInfDiseases, 2009 [6]	One of the following criteria: (1) an abscess or sinus tract communicating with the joint space, (2) positive preoperative aspiration culture on solid media, (3) ≥2 positive intraoperative cultures or 1 positive culture on solid media in conjunction with the presence of other indicators of infection (gross intracapsular purulence, elevated cell count and differential of the spirate fluid	30	94%	70%
Piper et al., PlosOne, 2010 [12]	≥1 of the following criteria: (1) visible pus surrounding the implant, (2) a positive histopathological examination, (3) a sinus tract communicating with the implant, or (4) positive periprosthetic tissue culture and positive sonicate fluid culture for the same microorganism	Knee 19 Hip 13	89% 82%	74% 60%
Alijanipour et al., CORR, 2013 [14]	MSIS 2011	Knee 30 Hip 30	94% 95%	68% 71%
Elgeidi et al., Int Orthop, 2014 [15]	≥1 of the following criteria: (1) visible pus surrounding prosthesis, (2) sinus tract communicating to implant, (3) growth of bacteria on culture (≥2 intraoperative cultures or growth of a virulent organism in a single specimen)	45	82%	83%
Shahi et al., JBJS Am, 2017 [19]	MSIS 2014	30	73%	78%
Fu et al., JoA, 2019 [21]	MSIS 2011	30	33%	100%
Paziuk et al., JoA, 2020 [42]	MSIS (not mentioned)	46	78%	79%
Xiong et al., JOSR, 2019 [22]	MSIS 2011	30	73%	100%
Yu et al., BMC Musculoskeletal Disorders, 2020 [23]	MSIS 2011	22	63%	74%
Qin et al., JoA, 2020 [24]	MSIS 2014	41	64%	70%
Bin et al., JoA, 2020 [26]	MSIS 2011	31	77%	97%
Wu et al., JoA, 2020 [27]	MSIS 2014	29	70%	92%
Huang et al., Orthopaedic Surgery, 2021 [28]	MSIS 2014	30	81%	88%
Yang et al., Sci Rep, 2021 [29]	ICM 2018	36.5	70%	86%

**Table 3:** Comparison of the literature regarding serum white blood cell count (WBC), percentage of neutrophils (%N), neutrophils to lymphocytes ratio (NLR), and platelet count to mean platelet volume ratio (PC/mPV) in the diagnosis of periprosthetic joint infection.

	Infection Definition	Cut-Off (×10^9^ Cells/L)	Sensitivity	Specificity
Nilsdotter-Augustinsson et al. ActaOrtho 2007 [10]	Positive cultures together with the clinical evaluations by orthopedic surgeons	10	92%	60%
Bottner et al., JBJS Br, 2007 [11]	Based on findings of intraoperative culture and histology	6.2	70%	60%
Toossi et al., JoA, 2012 [35]	≥1 of the following criteria: (1) positive culture, (2) Intraoperative purulence, (3) draining sinus tract. Or3 of the following 4: (a) ESR ≥ 30 mm/h, (b) CRP ≥ 10 mg/L, (c) Synovial WCC ≥ 1760 cells/µL (chronic) or ≥10,700 cells/µL, (d) percentage of neutrophils ≥ 73% (chronic) or 89% (acute).	7.8	55%	66%
Glehr et al., CORR, 2013 [13]	MSIS 2011	5.48	91%	34%
Elgeidi et al., Int Orthop, 2014 [15]	≥1 of the following criteria: (1) visible pus surrounding prosthesis, (2) sinus tract communicating to implant, (3) growth of bacteria on culture (≥2 intraoperative cultures or growth of a virulent organism in a single specimen)	9.2	91%	76%
Randau et al., Plos One, 2014 [16]	≥1 of the following criteria: (1) purulent synovial fluid or ≥1700 leukocytes/µL or ≥65% neutrophils in the joint aspirate (TKA) (≥3600 leukocytes/µL or ≥80% neutrophils (THA)), (2) histological confirmation of PJI, (3) pathogen detection in sterile joint aspiration or in at least two intraoperative tissue specimen, or (4) definitive signs of PJI clinically or intraoperatively (e.g., sinus tract)	10.3	21%	94%
Yuan et al., Surgical Infection, 2015 [17]	≥1 of the following criteria: (1) ≥1 positive tissue culture, (2) pus surrounding the prosthesis, or (3) positive histopathology	10.5	64%	54%
Klim et al., Sci Rep, 2018 [20]	MSIS 2011	5.68	90%	39%
Yu et al., BMC Musculoskeletal Disorders, 2020 [23]	MSIS 2011	8.91	35%	94%
Klim et al., Int Orthop, 2020 [25]	MSIS 2011	8.17	42%	92%
Yang et al., Sci Rep, 2021 [29]	ICM 2018	7.4	79%	49%
Sigmund et al., Int Orthop, 2021 [2]	EBJIS	10	36%	89%
**%N**		**Cut-Off**		
Toossi et al., JoA, 2012 [35]	≥1 of the following criteria: (1) positive culture, (2) Intraoperative purulence, (3) draining sinus tract. Or3 of the following 4: (a) ESR ≥ 30 mm/h, (b) CRP ≥ 10 mg/L, (c) Synovial WCC ≥ 1760 cells/µL (chronic) or ≥10,700 cells/µL, (d) percentage of neutrophils ≥ 73% (chronic) or 89% (acute).	68%	52%	75%
Sigmund et al., Int Orthop, 2021 [2]	EBJIS	69%	66%	67%
**NLR**		**Cut-Off**		
Yu et al., BMC Musculoskeletal Disorders, 2020 [23]	MSIS 2011	2.13	85%	68%
Sigmund et al., Int Orthop, 2021 [2]	EBJIS	3.82	63%	73%
**PC/mPV**		**Cut-Off**		
Paziuk et al., JoA, 2020 [42]	MSIS (year not mentioned)	31.7	48%	81%
Huang et al., Orthopaedic Surgery, 2021 [28]	MSIS 2014	31.7	55%	81%
Sigmund et al., Int Orthop, 2021 [2]	EBJIS	29.4	43%	81%

**Table 4:** Comparison of the literature regarding serum fibrinogen in the diagnosis of periprosthetic joint infection.

Fibrinogen	Infection Definition	Cut-Off (mg/dL)	Sensitivity	Specificity
Klim et al., Sci Rep, 2018 [20]	MSIS 2011	519	90%	66%
		574	81%	75%
Klim et al., Int Orthop, 2020 [25]	MSIS 2011	515	94%	73%
Bin et al., JoA, 2020 [26]	MSIS 2011	360	79%	95%
Wu et al., JoA, 2020 [27]	MSIS 2014	361	76%	86%
Sigmund et al., Int Orthop, 2021 [2]	EBJIS	457	69%	89%
Huang et al., Orthopaedic Surgery, 2021 [28]	MSIS 2014	401	78%	88%
Yang et al., Sci Rep, 2021 [29]	ICM 2018	420	86%	90%

**Table 5:** Comparison of the literature regarding plasma/serum D-dimer *n* in the diagnosis of periprosthetic joint infection.

D-Dimer	Infection Definition	Cut Off (ng/mL)	Sensitivity	Specificity	Serum/Plasma
Shahi et al., JBJS Am, 2017 [19]	MSIS 2014	850	89%	93%	not mentioned
Fu et al., JoA, 2019 [21]	MSIS 2011	850	67%	60%	Plasma
Xiong et al., JOSR, 2019 [22]	MSIS 2011	756	81%	80%	Serum
Pannu et al., JoA, 2020 [77]	ICM 2013	850	96%	32%	Serum
Qin et al., JoA, 2020 [24]	MSIS 2014	1170	93%	75%	Serum
Wu et al., JoA, 2020 [27]	MSIS 2014	410	76%	67%	Plasma
Huang et al., Orthopaedic Surgery, 2021 [28]	MSIS 2014	1170	60%	85%	Plasma

**Table 6:** Comparison of the literature regarding serum interleukin 6 (IL-6) in the diagnosis of periprosthetic joint infection.

Il-6	Infection Definition	Cut-Off (pg/mL)	Sensitivity	Specificity
Bottner et al., JBJS Br, 2007 [11]	Based on findings of intraoperative culture and histology	12	95%	87%
Glehr et al., CORR, 2013 [13]	MSIS 2011	2.55	94%	53%
Gollwitzer et al., JBJS Am, 2013 [80]	≥1 of the following criteria: (1) sinus tract, (2) 2 positive major criteria (positive intraoperative microbiological culture and positive histopathological analysis), or (3) 1 positive major criterion and 1 positive minor criterion (CRP > 1.0 mg/dL and/or positive microbiological culture of the aspirate)	1.89	47%	95%
Elgeidi et al., Int Orthop, 2014 [15]	≥1 of the following criteria: (1) visible pus surrounding prosthesis, (2) sinus tract communicating to implant, (3) growth of bacteria on culture (≥2 intraoperative cultures or growth of a virulent organism in a single specimen)	10.4	100%	93%
Randau et al., Plos One, 2014 [16]	≥1 of the following criteria: (1) purulent synovial fluid or ≥1700 leukocytes/µL or ≥65% neutrophils in the joint aspirate (TKA) (≥3600 leukocytes/µL or ≥80% neutrophils (THA)), (2) histological confirmation of PJI, (3) pathogen detection in sterile joint aspiration or in at least two intraoperative tissue specimen, or (4) definitive signs of PJI clinically or intraoperatively (e.g., sinus tract)	2.6 6.6	79% 49%	58% 88%
Ettinger et al., CID, 2015 [18]	MSIS 2011	5.12	80%	88%
Yu et al., BMC Musculoskeletal Disorders, 2020 [23]	MSIS 2011	8.07	80%	76%
Klim et al., Int Orthop, 2020 [25]	MSIS 2011	5.7	77%	70%

**Table 7:** Comparison of the literature regarding procalcitonin in the diagnosis of periprosthetic joint infection.

Procalitonin	Infection Definition	Cut-Off (ng/mL)	Sensitivity	Specificity
Bottner et al., JBJS Br, 2007 [11]	Based on findings of intraoperative culture and histology	0.3	33%	98%
Glehr et al., CORR, 2013 [13]	MSIS 2011	0.35 0.75	90% 48%	33% 100%
Randau et al., Plos One, 2014 [16]	≥1 of the following criteria: (1) purulent synovial fluid or ≥1700 leukocytes/µL or ≥65% neutrophils in the joint aspirate (TKA) (≥3600 leukocytes/µL or ≥80% neutrophils (THA)), (2) histological confirmation of PJI, (3) pathogen detection in sterile joint aspiration or in at least two intraoperative tissue specimen, or (4) definitive signs of PJI clinically or intraoperatively (e.g., sinus tract)	0.46	13%	100%
Yuan et al., Surgical Infection, 2015 [17]	≥1 of the following criteria: (1) ≥1 positive tissue culture, (2) pus surrounding the prosthesis, or (3) positive histopathology	0.5	80%	74%
Ettinger et al., CID, 2015 [18]	MSIS 2011	0.25	90%	28%
Klim et al., Int Orthop, 2020 [25]	MSIS 2011	0.1	40%	90%

**Table 8:** Summary.

Serum Parameter	Cut-Off	Sensitivity	Specificity
C-reactive Protein (CRP)	3–32 mg/L	62–100%	64–96%
Erythrocyte sedimentation rate (ESR)	13–46 mm/h	33–95%	60–100%
White blood cell count (WBC)	5.48–10.5 × 10^9^ cells/L	21–42%	89–94%
Percentage of neutrophils (%N)	68–69%	52–66%	67–75%
Neutrophils to lymphocytes ratio (NLR)	2.13–3.82	63–85%	68–73%
Platelet count to mean platelet volume ratio (PC/mPV)	29.4–31.7	43–55%	81%
Fibrinogen	360–574 mg/dL	69–94%	66–95%
D-dimer	410–1170 ng/mL	60–96%	32–93%
Interleukin 6 (IL-6)	1.89–12 pg/mL	47–100%	53–95%
Procalcitonin (PCT)	0.1–0.75 ng/mL	13–90%	28–100%
